# Neutrophils from critically ill septic patients mediate profound loss of endothelial barrier integrity

**DOI:** 10.1186/cc13049

**Published:** 2013-10-07

**Authors:** Elizabeth D Fox, Daithi S Heffernan, William G Cioffi, Jonathan S Reichner

**Affiliations:** 1 Division of Surgical Research, Department of Surgery, Alpert Medical School of Brown University, Rhode Island Hospital, Providence RI, USA; 2 Division of Trauma and Surgical Critical Care, Department of Surgery, Alpert Medical School of Brown University, Rhode Island Hospital, Providence RI, USA

## Abstract

**Introduction:**

Sepsis is characterized by systemic immune activation and neutrophil-mediated endothelial barrier integrity compromise, contributing to end-organ dysfunction. Studies evaluating endothelial barrier dysfunction induced by neutrophils from septic patients are lacking, despite its clinical significance. We hypothesized that septic neutrophils would cause characteristic patterns of endothelial barrier dysfunction, distinct from experimental stimulation of normal neutrophils, and that treatment with the immunomodulatory drug β-glucan would attenuate this effect.

**Methods:**

Blood was obtained from critically ill septic patients. Patients were either general surgery patients (Primary Sepsis (PS)) or those with sepsis following trauma (Secondary Sepsis (SS)). Those with acute respiratory distress syndrome (ARDS) were identified. Healthy volunteers served as controls. Neutrophils were purified and aliquots were untreated, or treated with fMLP or β-glucan. Endothelial cells were grown to confluence and activated with tissue necrosis factor (TNF)-α . Electric Cell-substrate Impedance Sensing (ECIS) was used to determine monolayer resistance after neutrophils were added. Groups were analyzed by two-way analysis of variance (ANOVA).

**Results:**

Neutrophils from all septic patients, as well as fMLP-normal neutrophils, reduced endothelial barrier integrity to a greater extent than untreated normal neutrophils (normalized resistance of cells from septic patients at 30 mins = 0.90 ± 0.04; at 60 mins = 0.73 ± 0.6 and at 180 mins = 0.56 ± 0.05; *p < 0.*05 vs normal). Compared to untreated PS neutrophils, fMLP-treated PS neutrophils caused further loss of barrier function at all time points; no additive effect was noted in stimulation of SS neutrophils beyond 30 min. Neutrophils from ARDS patients caused greater loss of barrier integrity than those from non-ARDS patients, despite similarities in age, sex, septic source, and neutrophil count. Neutrophils obtained after resolution of sepsis caused less barrier dysfunction at all time points. β-glucan treatment of septic patients’ neutrophils attenuated barrier compromise, rendering the effect similar to that induced by neutrophils obtained once sepsis had resolved.

**Conclusions:**

Neutrophils from septic patients exert dramatic compromise of endothelial barrier integrity. This pattern is mimicked by experimental activation of healthy neutrophils. The effect of septic neutrophils on the endothelium depends upon the initial inflammatory event, correlates with organ dysfunction and resolution of sepsis, and is ameliorated by β-glucan.

## Introduction

Neutrophils typify the early inflammatory response, accumulating at sites of infection and tissue injury [1,2]. Endothelial cells of the microvasculature represent a critical site of barrier regulation, selectively permitting passage of fluid, macromolecules, and cells into the extravascular tissue. Through a complex series of interactions involving binding of selectins, integrins, and adhesion molecules, the neutrophil rolls, arrests, and migrates through the endothelial barrier [3-6]. Increased intercellular space between endothelial cells permits paracellular transmigration of the neutrophil [3,7]. This process allows the neutrophil to follow a chemotactic gradient to a site of infection.

In sepsis, infection elicits a systemic inflammatory response. It has been estimated that more than 750,000 cases of severe sepsis occur in the United States each year [8]. Numerous processes have been implicated in the pathogenesis of sepsis and have served as targets for therapeutic trials, including cytokine mediators of the inflammatory response (tissue necrosis factor-α (TNFα)), immune receptors (interleukin-1 receptor, Toll-like receptor 4), and bacterial products (endotoxin); unfortunately, no target has yet been found to provide a mortality benefit in humans [3,9-12].

Sepsis-associated mortality has been shown to increase with the severity of sepsis [13]. In its severe form, sepsis is accompanied by evidence of organ dysfunction, with mortality reported to be 28 to 50% [8,14]. It is believed that neutrophils play a critical role in the development of organ failure. Their sequestration in capillary beds can lead to microvascular occlusion and subsequent tissue ischemia [3]. Additionally, neutrophils release a variety of substances, including reactive oxygen species and proteolytic enzymes [2,5,15], and these substances are known to affect endothelial barrier integrity [7]. With systemic activation of the immune system, neutrophils accumulate in vascular beds remote from the site of infection or tissue injury [10]; the widespread release of these substances is thought to contribute to the tissue damage associated with end-organ dysfunction such as acute lung injury or Acute Respiratory Distress Syndrome (ARDS) [10,16-18].

Following initial survival from septic events, much of the ensuing morbidity and mortality is driven by the prolonged effects of endothelial leak and end-organ failure [19,20]. Despite the clear clinical relevance of barrier function to morbidity and mortality in sepsis, no studies have yet examined the effect of neutrophils obtained from septic patients on endothelial monolayer integrity. Models of *in vitro* neutrophil stimulation exist, such as treatment with N-formyl-l-methionyl-l-leucyl-l-phenylalanine (fMLP); however, models of neutrophil stimulation, as well as animal models of sepsis, are unable to fully incorporate the complex environment of the septic patient, which includes active management and resuscitation along with comorbidities that can play a causative role in the development of sepsis.

We sought to characterize the pattern of endothelial barrier dysfunction induced by neutrophils from critically ill septic patients, with the hypothesis that these neutrophils would affect endothelial barrier integrity differently to both unstimulated and fMLP-stimulated neutrophils from healthy volunteers. Furthermore, prior work from our laboratory showed that endothelial barrier dysfunction caused by fMLP-stimulated neutrophils obtained from healthy donors could be ameliorated by treatment with soluble β-glucan [21]. β-Glucan is a ligand of the leukocyte integrin complement receptor 3 (CR3; CD11b/CD18) and is an immunomodulatory drug that has been studied in clinical trials as a therapeutic to reduce postoperative complications [22]. Whether soluble β-glucan could protect an endothelial monolayer from damage caused by neutrophils obtained from septic donors was determined in the current study.

Herein, we describe the novel findings that: the loss of endothelial barrier integrity induced by fMLP stimulation of neutrophils from healthy volunteers mimics barrier dysfunction induced by neutrophils from septic patients; neutrophils from patients who develop sepsis after traumatic injury are maximally activated with respect to their effect upon barrier function; barrier dysfunction is exacerbated in the presence of neutrophils from septic patients with ARDS; resolution of sepsis is characterized by improved barrier function; and treatment of neutrophils with pharmaceutical-grade β-glucan attenuates the barrier-altering effects of septic patient neutrophils, rendering their effect upon barrier function similar to that induced by neutrophils obtained once sepsis has resolved.

## Materials and methods

### Reagents

Pharmaceutical-grade soluble β-glucan (Imprime PGG®) was obtained from Biothera (Eagan, MN, USA). The β-glucan preparation contained <0.02% protein, <0.01% mannan, and 1% glucosamine. Lyophilized thrombin from human plasma, Histopaque 1077, l-cysteine, and dextran (~80 to 120 kDa molecular mass) were obtained from Sigma Life Sciences (St Louis, MO, USA). Rat-tail type I collagen was obtained from BD Biosciences (Bedford, MA, USA). Recombinant human TNFα was obtained from R&D Systems (Minneapolis, MN, USA). Trypsin and endothelial growth medium (EGM-2), containing SingleQuots® supplements, were purchased from Lonza (Walkersville, MD, USA). Human umbilical vein endothelial cells (HUVEC) were obtained from Cambrex (Walkersville, MD, USA). Electric cell-substrate impedance sensing (ECIS) cultureware electrode arrays (8W10E+) and a 16-well array station were obtained from Applied BioPhysics (Troy, NY, USA). All reagents used contained <0.1 pg/ml endotoxin as determined by Limulus amebocyte lysate screening (Lonza).

### Patient enrollment

This study was approved by the Institutional Review Board of Rhode Island Hospital. Written informed consent to participate and report results was provided by the patients participating in this study, or their surrogates. Critically ill septic patients in the surgical ICU and the trauma ICU of our institution were prospectively enrolled. Septic patients were identified as those fulfilling two or more systemic inflammatory response syndrome criteria with a clinically or microbiologically confirmed source of infection. We used standard systemic inflammatory response syndrome criteria, namely two or more of the following: heart rate >90 beats/minute; temperature <36°C or >38°C; respiratory rate >20 breaths/minute or PaCO_2_ <32 mmHg, or need for mechanical ventilation; white blood cell count <4,000 cells/mm^3^ or >12,000 cells/mm^3^, or >10% bands [23]. Patients were diagnosed with sepsis based on either microbiological data or direct inspection, such as perforated bowel at laparotomy. Pneumonia is routinely diagnosed at our institution using bronchoalveolar lavage, wherein only patients with a Clinical Pulmonary Infection Score ≥6 and >100,000 colony-forming units/ml on bronchoalveolar lavage are diagnosed with pneumonia. Abdominal sepsis was confirmed with either microbiology of drained intra-abdominal abscess or *Clostridium difficile*, or at laparotomy wherein a perforated viscus with purulent peritonitis was visualized. No sepsis was diagnosed based on clinical suspicion alone. Critically ill septic patients in the surgical ICU and the trauma ICU of our institution were prospectively enrolled within 24 hours of the diagnosis of sepsis.

Patients were categorized as either primary sepsis (PS) or secondary sepsis (SS). PS patients were nontrauma general surgery patients with a diagnosis of sepsis. SS patients were those who were initially admitted following traumatic injury and subsequently developed sepsis during the same hospitalization. Acute lung injury at the time of participation was defined according to the American–European Consensus Conference definition as a partial pressure of oxygen in arterial blood:inspired oxygen fraction ratio <300 mmHg, along with bilateral pulmonary infiltrates on chest radiograph in patients without clinical evidence of left atrial hypertension [24]. Patients were determined to have resolution of sepsis when they met fewer than two systemic inflammatory response syndrome criteria and had undergone treatment for their source of sepsis, including antibiotics for pneumonia and source control for intra-abdominal infections.

Charts were reviewed for demographics, including age and sex. Radiographs were evaluated for evidence of pulmonary infiltrates supporting the diagnosis of ARDS. Laboratory data including white blood cell count, neutrophil count, and arterial blood gases on the day of study participation were analyzed. In addition, charts and microbiological data were reviewed to identify the source of sepsis. Data extracted allowed for the calculation of the Acute Physiology and Chronic Health Evaluation II score at the time of the blood draw. Patients were excluded from enrollment if they had received any medication known to alter neutrophil functioning such as corticosteroids.

### Neutrophil isolation

Up to 15 ml blood were collected in sterile tubes containing ethylenediamine tetraacetic acid and were processed within 30 minutes of collection without storage. Neutrophils were isolated from whole blood using gradient centrifugation on Histopaque. Sedimentation of erythrocytes was performed using 3% dextran, and the neutrophil-rich supernatant then underwent hypotonic lysis of residual erythrocytes, yielding a >95% pure neutrophil population of >90% viability by trypan dye exclusion. Neutrophils were resuspended in Hanks Balanced Salt Solution minus calcium and magnesium, and were counted.

To assess whether the mere presence of the neutrophils induced a change in endothelial barrier function or whether the neutrophils needed to be functional, an aliquot of purified neutrophils underwent fixation with 1% paraformaldehyde for 20 minutes at room temperature. Cells were then washed twice with Hanks Balanced Salt Solution minus calcium and magnesium, resuspended and counted as previously described.

### β-Glucan treatment

For β-glucan treatment, purified neutrophils were incubated with 10 μg/ml of soluble pharmaceutical-grade β-glucan for 20 minutes at 37°C prior to their use in barrier function assays. This concentration was based on previous work by our group [21]. Dextran, a polysaccharide of similar molecular weight to β-glucan, was used as a negative control.

### Endothelial cell culture

HUVEC were grown on gelatin-coated flasks in EGM-2 medium. Cells were incubated at 37°C with 5% CO_2_. Once confluent, endothelial cells were trypsinized, counted, and used in barrier function assays. HUVEC were used within the first five passages.

### Measurement of endothelial barrier function

ECIS technology allows determination of changes in the impedance to current flow across an electrode in real time [25]. Wells of sterile 8W10E+ electrode arrays were reduced with 10 mM l-cysteine and coated with 30 μg/ml type I collagen. Endothelial cells were then plated and grown to confluence. Only wells with resistance of 800 to 1,800 Ohm were used in experiments. Initial experiments were conducted in the presence and absence of 20 ng/ml TNFα stimulation of endothelial cells for a period of 3 hours prior to the addition of neutrophils, in order to evaluate the effect of TNFα on neutrophil-induced changes in barrier function. All subsequent experiments, including those evaluating patient samples, were conducted using TNFα-activated monolayers. fMLP was added to a subset of wells (final concentration 10^–6^ M) immediately prior to addition of neutrophils. Resistance measurements were collected at 4,000 Hz. Neutrophil viability did not diminish through the 180-minute duration of the experiments as determined by trypan blue exclusion and by Wright staining for nuclear morphology indicative of apoptosis. Thrombin (0.8 units/ml), a molecule known to cause immediate loss of monolayer integrity, served as a positive control. EGM-2 medium served as a negative control. Finally, to assess whether changes in barrier function were dependent upon functional neutrophils, endothelial resistance was measured using paraformaldehyde fixed neutrophils. All experiments were carried out at 37°C with 5% carbon dioxide.

### Data analysis

Data are reported as mean normalized monolayer resistance ± standard error of the mean. Normalization was performed relative to the immediate pretreatment resistance of the well. Statistical analysis of patient characteristics was performed using Student’s *t* test for continuous data and Fisher’s exact test for categorical data. Evaluation of barrier function assays was performed using two-way analysis of variance with Holm–Sidak *post-hoc* analysis. Paired samples were analyzed using two-way repeated-measures analysis of variance. Statistical significance was defined as *P* <0.05.

## Results

### TNFα activation increases the susceptibility of endothelial cells to loss of barrier function

Using the ECIS system, we observed changes in transendothelial electrical resistance, thereby monitoring changes in monolayer integrity. Preliminary experiments evaluated the effect of TNFα activation of endothelial cells. Neutrophils obtained from healthy volunteers caused a decrease in the barrier function of HUVEC both in the absence and the presence of TNFα activation; this effect was greatest for activated endothelial cells (normalized resistance: at 30 minutes, 0.604 ± 0.02 vs. 0.767 ± 0.07, *P* <0.001; and at 60 minutes, 0.565 ± 0.01 vs. 0.765 ± 0.01, *P* <0.001). Given that activated endothelium more closely mimics the state of the endothelium in septic patients [26], all subsequent experiments were performed using TNFα-activated endothelial cells.

### Barrier compromise induced by neutrophils from septic patients is mimicked by fMLP stimulation of neutrophils from healthy volunteers

**Figure 1:**
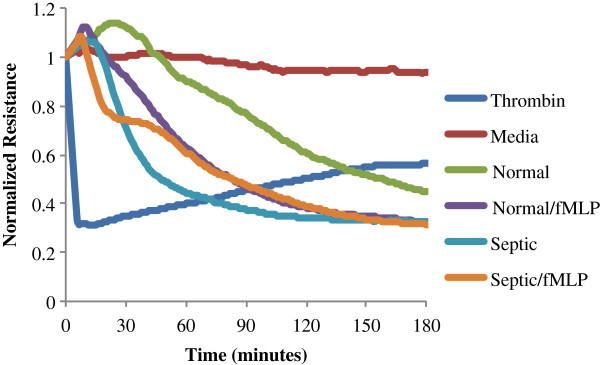
**Representative electric cell-substrate impedance sensing tracing.** Change in normalized monolayer resistance with respect to time. *t* = 0 represents the time of addition of thrombin, media, untreated neutrophils from a healthy volunteer (normal), N-formyl-l-methionyl-l-leucyl-l-phenylalanine (fMLP)-treated neutrophils from the same healthy volunteer (normal/fMLP), untreated neutrophils from a septic patient (septic), or fMLP-treated neutrophils from the same septic patient (septic/fMLP). fMLP treatment was undertaken using 10^–6^ M fMLP. All neutrophil populations were found to cause a progressive loss of barrier function.

Analyses were performed evaluating the effect of neutrophils obtained from all critically ill septic patients (*n* = 18) and neutrophils from healthy controls (*n* = 11); those from healthy volunteers were either exposed to activating doses of fMLP (10^–6^ M) or were left untreated. A representative ECIS tracing is provided in Figure 1. Treatment of endothelial cells with thrombin, a positive experimental control, led to an immediate loss of monolayer integrity. Wells containing media, which served as a negative control, did not demonstrate significant change in resistance over time. Endothelial barrier function was unchanged in the presence of paraformaldehyde-fixed neutrophils from both septic patients and healthy volunteers (data not shown), showing that barrier function compromise depends upon challenge with live neutrophils. All other neutrophil populations caused a progressive loss of endothelial barrier integrity. The magnitude of this effect varied with time, with source of the cells, and with fMLP treatment.

**Figure 2:**
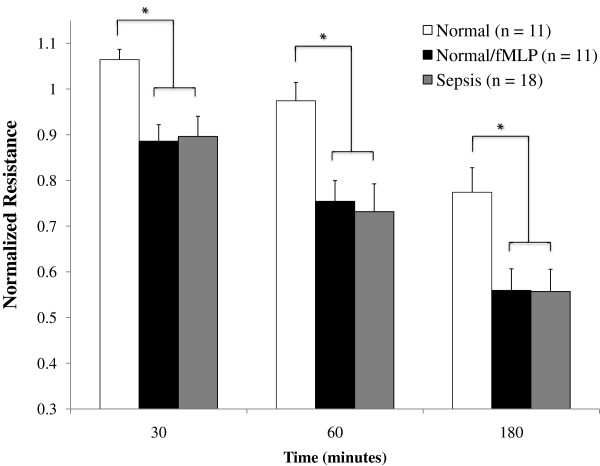
**Septic and normal/N-formyl-l-methionyl-l-leucyl-l-phenylalanine neutrophils induce similar patterns of barrier dysfunction.** Change in monolayer resistance at 30 minutes, 60 minutes, and 180 minutes. Groups were analyzed using two-way analysis of variance with Holm–Sidak *post-hoc* analysis. Values presented as mean ± standard error of the mean. Both normal/N-formyl-l-methionyl-l-leucyl-l-phenylalanine (fMLP) and septic patient neutrophils caused a greater change in barrier function than untreated normal neutrophils; however, there was no difference between normal/fMLP and septic patient neutrophils at any time point. *P <0.05 vs. normal.

Comparisons of the effect of neutrophils from septic patients, normal controls and normal/fMLP neutrophils upon endothelial barrier function are shown in Figure 2. When compared with untreated normal neutrophils, it was noted that neutrophils from septic patients as well as fMLP-treated neutrophils from healthy donors induced significantly greater loss of endothelial barrier function. This effect was seen as early as 30 minutes after the addition of neutrophils and persisted across all time points. It was noted that, at all time points, there was no statistically significant difference between septic patient neutrophils and fMLP-treated normal neutrophils in terms of their effect upon barrier function (normalized resistance: at 30 minutes, 0.90 ± 0.04 vs. 0.89 ± 0.04, *P* = 0.37; at 60 minutes, 0.73 ± 0.06 vs. 0.75 ± 0.05, *P* = 0.57; and at 180 minutes, 0.56 ± 0.05 vs. 0.56 ± 0.05, *P* = 0.73).

### Neutrophils from patients with primary sepsis, but not secondary sepsis, can be further stimulated at all time points

We sought to determine whether the occurrence of a second clinical inflammatory hit (the development of sepsis following trauma) influenced neutrophil-induced endothelial barrier dysfunction. Septic patients were designated as either PS (*n* = 12) or SS (*n* = 6) (Table 1). There was no difference between groups with respect to age (59.9 ± 7.6 years vs. 44 ± 7.8 years, *P* = 0.2), male sex (58% vs. 50%, *P* = 0.9) or Acute Physiology and Chronic Health Evaluation II score (11 ± 0.9 vs. 12.4 ± 1.1, *P* = 0.36), and in both groups the source of sepsis was either intra-abdominal or pneumonia, with abdominal sepsis being the predominant source. Additional clinical parameters are shown in Table 1. Untreated neutrophils from PS patients caused a similar effect upon barrier function when compared with untreated neutrophils from SS patients at all time points (normalized resistance: at 30 minutes, 0.94 ± 0.07 vs. 0.85 ± 0.03, *P* = 0.4; at 60 minutes, 0.75 ± 0.08 vs. 0.69 ± 0.09, *P* = 0.6; at 180 minutes, 0.57 ± 0.06 vs. 0.53 ± 0.08, *P* = 0.8). The average time to onset of the second hit was 5 days.

**Table 1:** Characteristics of septic patients

**Primary sepsis vs. secondary sepsis**	**Primary sepsis**	**Secondary sepsis**	***P *** **value**
	**(*n *** **= 12)**	**(*n *** **= 6)**	
Age (years)	59.9 ± 7.6	44 ± 7.8	0.2
Male sex (%)	58	50	0.9
Source of sepsis (%)			
Abdominal	58	67	0.9
Pneumonia	42	33	0.9
White cell count (×10^9^ cells/l)	15.1 ± 1.1	18.1 ± 1.9	0.2
Neutrophil count (×10^9^ cells/l)	12.2 ± 0.9	12.6 ± 1.4	0.8
**Septic patients with ARDS compared with those without evidence of ARDS**	**ARDS**	**No ARDS**	***P *** **value**
	**(*n *** **= 6)**	**(*n *** **= 12)**	
Age (years)	57.5 ± 8.9	54.1 ± 6.7	0.8
Male sex (%)	50	58	0.9
Source of sepsis (%)			
Abdominal	67	58	0.9
Pneumonia	33	42	0.9
White cell count (×10^9^ cells/l)	16.9 ± 2	15.7 ± 1.1	0.6
Neutrophil count (×10^9^ cells/l)	13.5 ± 1	11.8 ± 1	0.3

Characteristics listed are those on the day of study participation. Age, white blood cell count, and neutrophil count were analyzed using Student’s *t* test, and male sex and source of sepsis were analyzed using Fisher’s exact test. There were no differences between comparison groups. ARDS, acute respiratory distress syndrome.

**Figure 3:**
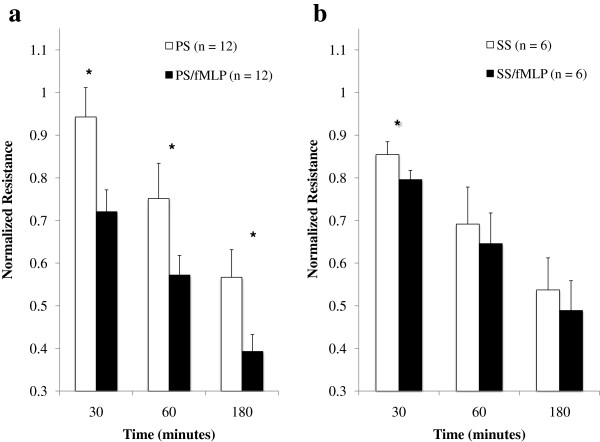
**N-formyl-l-methionyl-l-leucyl-l-phenylalanine treatment of primary sepsis leads to more profound loss of barrier function.** Change in monolayer resistance at 30 minutes, 60 minutes, and 180 minutes. Groups were analyzed using two-way repeated-measures analysis of variance with Holm–Sidak *post-hoc* analysis. Values presented as mean ± standard error of the mean. Whereas primary sepsis (PS) neutrophils could be further stimulated at every time point **(a)**, this effect was only present at 30 minutes for secondary sepsis (SS) neutrophils; no difference was noted thereafter **(b)**. *P <0.05. fMLP, N-formyl-l-methionyl-l-leucyl-l-phenylalanine.

fMLP was used to interrogate neutrophils from both groups of patients, because *in vitro* fMLP treatment of maximally stimulated cells would not be expected to lead to enhanced barrier dysfunction. Following treatment with fMLP, neutrophils from PS patients were noted to cause a further decrease in barrier function at all time points (normalized resistance: at 30 minutes, 0.72 ± 0.05 vs. 0.94 ± 0.07, *P* <0.001; at 60 minutes, 0.57 ± 0.05 vs. 0.75 ± 0.08, *P* <0.001; and at 180 minutes, 0.39 ± 0.04 vs. 0.57 ± 0.06, *P* <0.001) (Figure 3a). However, fMLP treatment of SS neutrophils led to greater loss of barrier integrity only at 30 minutes (normalized resistance: 0.80 ± 0.02 vs. 0.85 ± 0.03, *P* = 0.04); there was no difference at other time points (normalized resistance: at 60 minutes, 0.65 ± 0.07 vs. 0.69 ± 0.09, *P* = 0.1; and at 180 minutes, 0.49 ± 0.07 vs. 0.53 ± 0.08, *P* = 0.09) (Figure 3b).

### Neutrophils from septic patients with ARDS display even greater capacity for endothelial barrier compromise

**Figure 4:**
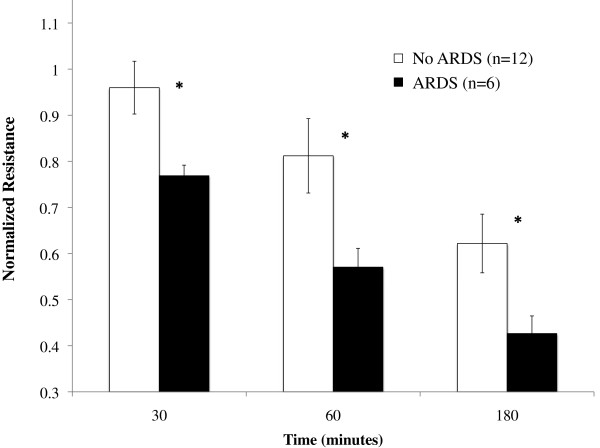
**Neutrophils from acute respiratory distress syndrome patients induce greater endothelial barrier dysfunction.** Change in monolayer resistance at 30 minutes, 60 minutes, and 180 minutes. Groups were analyzed using two-way analysis of variance with Holm–Sidak *post-hoc* analysis. Values presented as mean ± standard error of the mean. Neutrophils from septic patients with acute respiratory distress syndrome (ARDS) produced greater loss of barrier function than those obtained from septic patients without ARDS. *P <0.05.

Patients meeting the definition of ARDS at the time of study participation were identified. There was no difference between patients with ARDS (*n* = 6) and those without ARDS (*n* = 12) with respect to age (57.5 ± 8.9 years vs. 54.1 ± 6.7 years, *P* = 0.8), male sex (50% vs. 58%, *P* = 0.9), abdominal source of sepsis (67% vs. 58%, *P* = 0.9) or Acute Physiology and Chronic Health Evaluation II score (11.6 ± 0.99 vs. 11.0 ± 1.3, *P* = 0.9) Additionally, there was no difference between ARDS and non-ARDS patients in terms of total white blood cell count (16.9 ± 2 vs. 15.7 ± 1.1, *P* = 0.6) and neutrophil count (13.5 ± 1 ×10^9^ cells/l vs. 11.8 ± 1 ×10^9^ cells/l, *P* = 0.3) (Table 1). The effect of neutrophils obtained from septic patients with ARDS was compared with that of neutrophils obtained from septic patients without ARDS. At each time point, neutrophils from ARDS patients induced significantly greater loss of endothelial barrier integrity than those from patients without ARDS (normalized resistance: at 30 minutes, 0.77 ± 0.02 vs. 0.96 ± 0.06, *P* = 0.045; at 60 minutes, 0.57 ± 0.04 vs. 0.81 ± 0.08, *P* = 0.012; and at 180 minutes, 0.43 ± 0.04 vs. 0.62 ± 0.06, *P* = 0.040) (Figure 4).

### Resolution of sepsis is paralleled by improvement in endothelial barrier function

**Figure 5:**
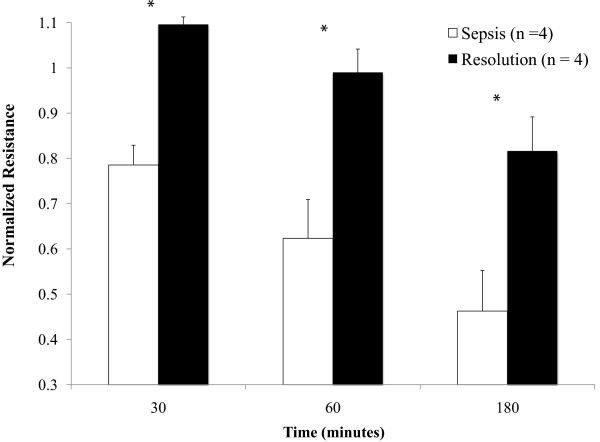
**Resolution of sepsis leads to improved barrier function.** Change in monolayer resistance at 30 minutes, 60 minutes, and 180 minutes. Groups were analyzed using two-way repeated-measures analysis of variance with Holm–Sidak *post-hoc* analysis. Values presented as mean ± standard error of the mean. The barrier-altering effect of neutrophils obtained once sepsis had resolved was significantly less than the effect of those obtained from the same patient during sepsis. *P <0.05.

Patients were followed over time as their septic physiology resolved. All patients found to have resolution of sepsis (*n* = 4) remained in the ICU at the time at which resolution of sepsis samples were obtained. A paired comparison of neutrophil-induced changes in barrier integrity was then undertaken. At all time points, neutrophils obtained once sepsis had resolved demonstrated less alteration of barrier function than neutrophils obtained when the same patient was septic (normalized resistance: at 30 minutes, 1.10 ± 0.02 vs. 0.76 ± 0.04, *P* = 0.048; at 60 minutes, 0.99 ± 0.05 vs. 0.62 ± 0.09, *P* = 0.029; and at 180 minutes, 0.82 ± 0.08 vs. 0.46 ± 0.09, *P* = 0.032) (Figure 5).

### Treatment of neutrophils with β-glucan ameliorates loss of barrier integrity caused by neutrophils from septic patients

**Figure 6:**
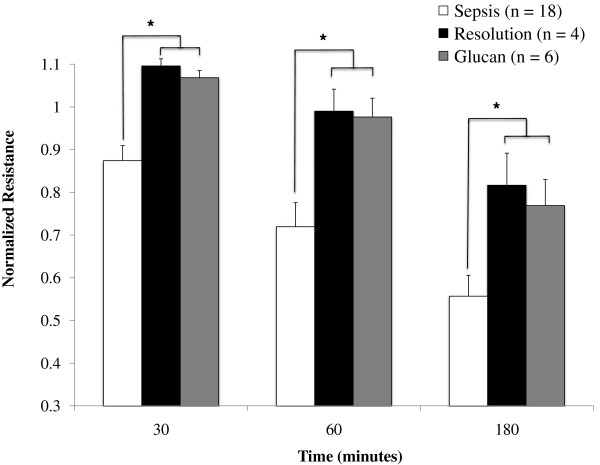
**β-Glucan attenuates loss of barrier dysfunction induced by neutrophils from septic patients.** Change in monolayer resistance at 30 minutes, 60 minutes, and 180 minutes comparing neutrophil samples from all patients with sepsis (sepsis), samples obtained once sepsis had resolved (resolution), and samples of septic patients’ neutrophils treated with β-glucan (glucan). Groups were analyzed using two-way analysis of variance with Holm–Sidak *post-hoc* analysis. Values presented as mean ± standard error of the mean. Treatment of neutrophils from septic patients with β-glucan led to an effect upon barrier function that was similar to that induced by neutrophils obtained upon resolution of sepsis; both groups caused less barrier dysfunction than untreated neutrophils from septic patients. *P <0.05 vs. sepsis.

To investigate the potential therapeutic role of β-glucan, purified neutrophils from septic patients were treated with soluble β-glucan as described in Materials and methods*.* Their effect upon barrier function was compared with that induced by untreated neutrophils from septic patients, as well as untreated neutrophils from resolution of sepsis patients (Figure 6). Neutrophils treated with β-glucan (*n* = 6) caused less of an alteration in barrier function at all time points compared with untreated neutrophils from septic patients (normalized resistance: at 30 minutes, 1.07 ± 0.02 vs. 0.87 ± 0.04, *P* = 0.048; at 60 minutes, 0.98 ± 0.04 vs. 0.72 ± 0.06, *P* = 0.005; and at 180 minutes, 0.77 ± 0.06 vs. 0.56 ± 0.05, *P* = 0.018). As a comparison, treatment of patient neutrophils with dextran, a control polysaccharide of similar molecular weight to β-glucan, did not improve barrier function compared with untreated neutrophils from septic patients (data not shown). Additionally, throughout the course of the experiment, the effect of neutrophils treated with β-glucan was noted to resemble the effect of neutrophils obtained once sepsis had resolved (normalized resistance: at 30 minutes, 1.07 ± 0.02 vs. 1.10 ± 0.02, *P* = 0.8; at 60 minutes, 0.98 ± 0.04 vs. 0.99 ± 0.05, *P* = 0.9; and at 180 minutes, 0.77 ± 0.06 vs. 0.82 ± 0.08, *P* = 0.7) (Figure 6).

## Discussion

Increased endothelial barrier permeability, and the resultant microvascular leak, is known to become pathologic in the setting of sepsis. Prolonged effects of loss of endothelial barrier integrity can lead to edema, hypotension, and serious end-organ complications [15,20,27]. Therapeutic options aimed at limiting or reversing the effects of endothelial leak remain limited [28] and largely supportive.

This is the first study demonstrating the real-time quantitative loss of barrier function induced by neutrophils from critically ill septic patients. We show the kinetics to be similar to the loss of barrier integrity due to challenge from normal neutrophils stimulated *in vitro*, an effect that diminishes upon resolution of sepsis. Clinical differences among septic patients were found to correlate with distinct patterns of endothelial barrier dysfunction. Moreover, the therapeutic potential of pharmaceutical-grade β-glucan in protecting the endothelial monolayer from loss of barrier integrity by septic neutrophils is shown.

Studies of neutrophils and endothelial cells in humans have largely focused on soluble biomarkers [29,30], receptor expression [31,32], and functional assays such as phagocytosis and reactive oxygen species production [33]. Microvascular permeability has been found to be significantly increased in patients with severe sepsis, compared with critically ill nonseptic patients [34]. We show that septic patients’ neutrophils induce profound loss of barrier function, and that, counter to our original hypothesis, this effect is mimicked by experimental activation of normal neutrophils. Although an important but unexpected finding, we do recognize that this is only one aspect of neutrophil biology. As such, future work will continue to characterize further similarities and/or difference between *in vitro* fMLP stimulated and septic neutrophils with respect to receptor alterations, intracellular signaling pathways, and other functional aspects of these neutrophils. This remains especially important in light of numerous interventions that have shown promise in animal models but subsequently failed in clinical trials involving the complex physiologic environment [11,35] within the septic patient.

Our data address a salient component of the two-hit hypothesis [36], in which the immune system is noted to be altered by a stimulus such as traumatic injury. Both an exaggerated immune response and a state of immune paralysis have been described in the post-trauma setting. Clinical practices over the past several decades have been altered based on this understanding with improved patient outcomes. We wished to begin to explore a specific aspect of the immune system (neutrophils effect upon barrier function) between patients with primary infections versus the more complex inflammatory milieu of this second-hit infection. While we did not detect a difference in the barrier dysfunction induced by neutrophils from patients with PS compared with those with SS, we did reveal a marked difference in their ability to be further activated (shown in Figure 3). Specifically, we have demonstrated that neutrophils from patients with PS can be further stimulated with fMLP to produce a greater loss of endothelial barrier integrity at every time point. This was not the case for neutrophils from patients with SS. It is known that neutrophils obtained from traumatically injured patients exhibit enhanced superoxide generation [37], as well as impaired phagocytosis and reduced rates of apoptosis [38], indicating that traumatic injury itself can stimulate neutrophils. Superoxide generation and proinflammatory cytokine levels are further enhanced in those trauma patients who sustain an infectious complication [38]. Similar to our finding that neutrophils from SS patients showed no increase in effect upon barrier function following treatment with fMLP, Botha and colleagues have demonstrated that neutrophils from traumatically injured patients subjected to a second inflammatory stimulus (platelet-activating factor) could not be further stimulated to produce superoxide when treated with fMLP [37]. Further work is needed to clarify whether this represents a state of immune paralysis or maximal stimulation.

Although improved over the past decade, both sepsis and acute lung injury continue to have mortality rates >25% [8,14,39]. Sepsis itself is associated with pulmonary capillary permeability and pulmonary edema [40]; this is exacerbated in patients with ARDS, and correlates with mortality [41]. In a murine model of acute lung injury, reduction of endothelial cell contractility was associated with reduced pulmonary neutrophil infiltration, reduced pulmonary edema formation, and improved survival [42]. Additionally, it is known that neutrophils play a key role in the development of lung injury. Using a trauma-sepsis two-hit model, Perl and colleagues have shown that neutrophil depletion leads to a reduction in pulmonary infiltrates and an improvement in alveolar architecture [43]. Moreover, it has been shown that neutrophils in bronchoalveolar lavage fluid samples from patients with acute lung injury have elevated levels of the integrin subunit CD11b, compared with levels on circulating neutrophils [44]. These studies highlight the burden of neutrophil influx and excessive endothelial permeability in ARDS. We have shown that clinically evident microvascular hyperpermeability, as seen in ARDS, does indeed correlate with endothelial barrier dysfunction observed *in vitro*. Because our study evaluated only circulating neutrophils from ARDS patients, we would hypothesize that pulmonary neutrophils may demonstrate an even greater effect.

Our data demonstrate an intriguing new finding that clinical resolution of sepsis is paralleled by reduction in the endothelial barrier dysfunction induced by neutrophils from these patients. Previous work has demonstrated that changes in the degree of critical illness are accompanied by functional changes of circulating neutrophils. Kaufmann and colleagues studied neutrophils from patients with increasing severity of sepsis (sepsis, severe sepsis, and septic shock) and showed that these septic states can be distinguished on the basis of reduced fraction of neutrophils participating in phagocytosis of zymosan particles as well as alterations in peroxide generation [33]. Ibbotson and colleagues reported that neutrophils obtained from septic patients expressed high levels of α_4_ integrins compared with healthy volunteers; these levels were found to decrease as patients improved clinically [35]. Our study highlights the novel finding of differential effects exerted on the critically important point of endothelial barrier dysfunction. Our data therefore suggest a possible therapeutic target in limiting progression of the inflammatory state associated with sepsis. In this regard, normal healthy controls were used as a comparison because we believe that any potential therapeutic interventions that may be based on these findings will aim to restore the critically ill septic patients back to healthy normality. At this point, choosing nonseptic critically ill patients may only address a portion of the potential therapeutic opportunities.

Changes in endothelial barrier integrity have been shown to be related to neutrophil β_2_ integrin signaling, leading to changes in endothelial cytosolic free calcium and reorganization of actin filaments [1]. It has further been suggested that altered integrin function may be an important factor in neutrophil maldistribution remote from sites of infection in sepsis [45]. Engagement of β_2_ integrins causes release of substances such as heparin-binding protein, which is known to promote neutrophil adhesion to endothelial cells via upregulation of adhesion molecules such as intercellular adhesion molecule-1 [46], and to cause endothelial cytoskeletal reorganization [47]. CR3 (CD11b/CD18) is one such β_2_ integrin, known to participate in neutrophil adhesion and transmigration. This molecule contains two key structural domains: the I domain, which binds ligands such as intercellular adhesion molecules and fibrinogen; and the lectin-like domain, which binds microbial polysaccharides such as β-glucan. We have previously shown that co-occupancy of these two domains affects several neutrophil functions, leading to the augmentation of chemotaxis [48] and attenuation of transmigration [21].

β-Glucan has been extensively studied as an immunomodulatory agent [49,50]. The soluble form of β-glucan used here has been studied in a variety of clinical trials and is known to be well tolerated, eliciting few side effects [51], and is not associated with cytokine production [52]. Additionally, β-glucan administration has been shown to limit infectious complications in patients; in a randomized control trial, Dellinger and colleagues showed that perioperative administration of β-glucan was associated with a significant reduction in infectious morbidity and mortality in patients undergoing noncolorectal surgery [22].

Our laboratory has previously demonstrated that β-glucan treatment of fMLP-treated normal neutrophils protects endothelial barrier integrity [21]. This effect was found to occur secondary to binding of β-glucan to the lectin-like domain of CR3. This intervention did not alter neutrophil adhesion to activated endothelial cells; however, it did achieve significant retention of barrier integrity, which was paralleled by a reduction in neutrophil transmigration. We expand upon this prior work, demonstrating a protective effect of soluble β-glucan in neutrophils from septic patients. In the current study, we show that β-glucan treatment is associated with improved barrier integrity throughout the course of the experiment. Studies using sepsis models often aim to prevent activation of neutrophils [53]; however, sepsis is rarely an event that can be prevented. Rather, many septic events, such as pneumonia, perforated viscus, or rapidly progressive skin and soft tissue infections, present to clinicians in states of advanced physiological derangement. The use of neutrophils from septic patients mimics the clinical state in which such a therapeutic agent might be administered. Our data demonstrating that β-glucan treatment can limit the ability of septic patient neutrophils to compromise endothelial barrier integrity, thereby causing them to behave more like neutrophils from patients with resolved sepsis, introduces a potential therapeutic mechanism.

## Conclusions

Sepsis is marked by significant changes in neutrophil function, including the induction of endothelial barrier dysfunction. Clearly, some degree of endothelial permeability to neutrophils is beneficial to the host, allowing for bacterial containment and eradication; however, its prolonged and dysregulated effects can lead to serious morbidity and mortality. Endothelial barrier function represents a target with significant diagnostic and therapeutic potential; a comprehensive understanding of the effect of neutrophils from septic patients upon endothelial barrier function is critical and will depend upon further understanding of the characteristics of neutrophils obtained from septic patients, and particularly those with organ dysfunction. Given the differences between neutrophils obtained during sepsis and during its resolution, the phenotype of the neutrophil obtained once sepsis has resolved may represent a therapeutic target, and β-glucan treatment may serve as a model for future therapeutic development.

## Key messages

•Neutrophils obtained from septic patients mediate significant compromise of endothelial barrier function.

•fMLP treatment of neutrophils from healthy individuals can mimic this effect.

•Clinically evident vascular leak seen in ARDS is paralleled by experimentally-measurable endothelial barrier dysfunction.

•Resolution of sepsis correlates with attenuated loss of barrier integrity.

•β-Glucan treatment of septic patients’ neutrophils ameliorates endothelial barrier dysfunction and may serve as a model for development of therapeutics.

## Abbreviations

ARDS: Acute respiratory distress syndrome; CR3: Complement receptor 3; ECIS: Electric cell-substrate impedance sensing; fMLP: N-formyl-l-methionyl-l-leucyl-l-phenylalanine; HUVEC: Human umbilical vein endothelial cells; PS: Primary sepsis; SS: Secondary sepsis; TNF: Tissue necrosis factor.

## Competing interests

The authors declare that they have no competing interests.

## Authors’ contributions

All authors contributed to the experimental conception and design. EDF was responsible for patient enrollment, clinical and experimental data acquisition and analysis, and manuscript drafting. WG and DSH were involved in clinical and experimental data analysis, and manuscript drafting. JSR participated in experimental data analysis. All authors were involved in critical revision for intellectual content, and read and approved the final manuscript.
